# Transcriptome Analysis of the Melon-*Fusarium oxysporum* f. sp. *melonis* Race 1.2 Pathosystem in Susceptible and Resistant Plants

**DOI:** 10.3389/fpls.2017.00362

**Published:** 2017-03-17

**Authors:** M. Silvia Sebastiani, Paolo Bagnaresi, Sara Sestili, Chiara Biselli, Antonella Zechini, Luigi Orrù, Luigi Cattivelli, Nadia Ficcadenti

**Affiliations:** ^1^Research Unit for Vegetable Crops in Central Areas, Council for Agricultural Research and EconomicsAscoli Piceno, Italy; ^2^Genomics Research Centre, Council for Agricultural Research and EconomicsPiacenza, Italy

**Keywords:** RNA-Seq, plant-pathogen interaction, gene expression, fusarium wilt, melon, molecular mechanism

## Abstract

*Fusarium oxysporum* f. sp. *melonis* Snyd. & Hans race 1.2 (FOM1.2) is the most virulent and yield-limiting pathogen of melon (*Cucumis melo* L.) worldwide. Current information suggest that the resistance to race 1.2 is controlled by multiple recessive genes and strongly affected by the environment. RNA-Seq analysis was used to identify candidate resistance genes and to dissect the early molecular processes deployed during melon-FOM1.2 interaction in the resistant doubled haploid line NAD and in the susceptible genotype Charentais-T (CHT) at 24 and 48 h post-inoculation (hpi). The transcriptome analysis of the NAD-FOM1.2 interaction identified 2,461 and 821 differentially expressed genes (DEGs) at 24 hpi and at 48 hpi, respectively, while in susceptible combination CHT-FOM1.2, 882 and 2,237 DEGs were recovered at 24 hpi and at 48 hpi, respectively. The overall expression profile suggests a prompt activation of the defense responses in NAD due to its basal defense-related machinery that allows an early pathogen recognition. Gene Ontology (GO) enrichment analyses revealed a total of 57 GO terms shared by both genotypes and consistent with response to fungal infection. GO classes named “chitinase activity,” “cellulase activity,” “defense response, incompatible interaction,” “auxin polar transport” emerged as major factors of resistance to FOM1.2. The data indicated that NAD reacts to FOM1.2 with a fine regulation of Ca^2+^-mediated signaling pathways, cell wall reorganization, and hormone crosstalk (jasmonate and ethylene, auxin and abscissic acid). Several unannotated transcripts were recovered providing a basis for a further exploration of the melon resistance genes. DEGs belonging to the FOM1.2 genome were also detected *in planta* as a resource for the identification of potential pathogenicity factors. This work provides a broader view of the dynamic changes of the melon transcriptome triggered by FOM1.2 and highlights that the resistance response of NAD is mainly signaled by jasmonic acid and ethylene pathways mediated by ABA and auxin. The role of candidate plant and fungal responsive genes involved in the resistance is discussed.

## Introduction

With 31.9 million of tons produced worldwide melon is one of the most cultivated horticultural crop (http://faostat.fao.org; 2012). Furthermore, due to its high economic value, short generation time, relatively small genome, and highly polymorphic phenotypes, especially in vegetative and fruit morphology, melon is also considered an interesting model species (Ezura and Fukino, 2009). *Fusarium oxysporum* f. sp. *melonis* (FOM) (Leach & Currence) Snyd. & Hans causes fusarium wilt, one of the most destructive and less controllable diseases in melon. FOM can persist in the soil indefinitely due to the production of chlamydospores and to the colonization of plant residues including roots of non-susceptible crops cultivated in rotation (Zuniga et al., 1997). Risser et al. (1976) identified four races of FOM (0, 1, 2, and 1.2) on the basis of the host resistance genes overcome by variants of the fungus. *Fom-1* and *Fom-2*, two dominant independently-inherited resistance genes, provide long-lasting stable resistance to races 0, 2, and races 0, 1, respectively (Risser et al., 1976). In addition, a new recessive gene *fom-4*, linked to *Fom-1*, confers resistance to races 0, 2 in the *Tortuga* melon line (Oumouloud et al., 2010). Despite these sources of resistance, fusarium wilt remains a relevant disease mainly because of FOM race 1.2 (FOM1.2) that represents the most widespread and harmful race causing economic losses up to 100% of melon yield (Luongo et al., 2015). Resistance to race 1.2 is complex being controlled by multiple recessive genes and strongly affected by the environment (Perchepied and Pitrat, 2004). Several studies on different melon genotypes have identified nine QTLs (Perchepied et al., 2005), two complementary recessive genes and a major recessive QTL linked to a locus controlling fruit netting (Herman and Perl-Treves, 2007; Herman et al., 2008) as the main genetic factors controlling FOM1.2 resistance.

The doubled-haploid (DH) resistant line NAD, used in this work, was developed by parthenogenesis *in situ* technique from the resistant mother plant Isabelle (Ficcadenti et al., 1995). After repetitive artificial inoculations with FOM1.2 NAD showed a high level of resistance, greater than the resistance observed in the mother plant (Ficcadenti et al., 2002). The genetic analysis of the NAD resistance has not yet revealed all the genetic determinants involved in FOM1.2 resistance, although there are evidence supporting the presence of *Fom-2* (Sestili et al., 2002). Charentais- T (CHT) is a genotype extremely susceptible to all FOM races, despite it shares a high genetic similarity with the resistant line NAD as demonstrated by molecular analyses (Sestili et al., 2008, 2011a). Therefore, the NAD-CHT-FOM1.2 experimental system represents an ideal genetic tool to investigate the molecular and genetics basis of melon-FOM1.2 interaction. In resistant melon plants, FOM1.2 induces an early plant resistance response (within the first 2 days post inoculation) that acts by reducing the invading pathogen vitality and its development (Sestili et al., 2011b). In this scenario, the identification of genes involved in the resistance and the depiction of the molecular basis underlying plant-host interaction can represent an important achievement to develop new breeding control strategies against FOM1.2 in melon. The use of RNA-Sequencing (RNA-Seq) represents the most powerful tool for transcriptome characterization, allowing gene discovery and global gene expression profiling. Several plant-pathogen interactions have been studied by using RNA-Seq (Bagnaresi et al., 2012; Li et al., 2012; Zhu et al., 2013; Pombo et al., 2014) but, to the best of our knowledge, this approach has never been used to compare the defense responses in resistant vs. susceptible melon-FOM1.2 interaction. In the present study, RNA-Seq analysis was carried out to investigate the dynamic changes of melon transcriptome in response to FOM1.2 infection and to gain new insights on genes underlying the resistance mechanisms against this dreadful pathogen.

## Materials and methods

### Plant material and *in vivo* inoculations

The resistant DH line NAD, developed by parthenogenesis from irradiated pollen of the resistant mother plant Isabelle (Ficcadenti et al., 1995), and the susceptible cultivar CHT (*Cucumis melo* var. *cantalupensis*) were used. Seedlings were grown in greenhouse at 25 ± 2°C with 80–90% relative humidity in plastic pots filled with sterilized soil. The FOM1.2 strain ISPaVe1018 was obtained from the fungal collection of the Plant Pathology Research Centre (CREA-PAV, Rome). The preparation of the inoculum and the artificial inoculation procedures were reported in Sestili et al. (2011b). After root trimming, the plantlets were dipped for 30 min in the conidial suspension. Mock-inoculations were performed in sterile distilled water. Seedlings were transferred into plastic pots filled with sterilized soil and maintained in greenhouse at 25 ± 2°C with 80–90% relative humidity. The stem of 10 plants per each time point (24 and 48 h post inoculation) were pooled and used for the transcriptome analysis. In addition, 50 plants for each genotype were phenotypically screened at 8, 15, 21, and 35 days post inoculation (dpi).

### RNA preparation and illumina sequencing

Total RNA was isolated from infected and mock-treated plants at 24 and 48 h post inoculation (hpi) using the TRIzol reagent (Invitrogen) and purified by the Qiagen RNeasy® Minikit (Qiagen), according to manufacturer's instructions. Two biological replicates for each genotype and treatment were used according to recommended RNA-Seq standards (Encode project https://genome.ucsc.edu/ENCODE/protocols/dataStandards/ENCODE_RNAseq_Standards_V1.0.pdf, 2011). The RNA-Seq libraries were produced with 4 μg of total RNA following the instructions of the Illumina TruSeq RNA sample preparation kit (FC-122-1001). After amplification and purification, the libraries with an average size of 300 bp were checked on the 2% low range ultra-agarose gel (BIO-RAD). RNA quality (RIN > 8) and library size were assayed on a 2100 Bioanalyzer (Agilent Technologies). Libraries were sequenced on an Illumina Genome Analyzer IIx to produce 51 bp single-end reads. FastQ file generation was performed by CASAVA v1.8.2.

### Quantitative RT-PCR analysis

Thirteen differentially expressed genes (DEGs), characterized by interesting expression profiles in response to FOM1.2 infection in compatible and incompatible combination, were selectedqRT-PCR. First strand cDNAs was synthesized from 100 ng of total RNA using the High Capacity cDNA Reverse Transcription Kit (Applied Biosystem). Primers were designed using Primer3 Software (http://frodo.wi.mit.edu/primer3/; Table 1) and the specificity was checked by blasting their sequences in the NCBI database. The melon *RIBOSOMAL PROTEIN L2* constitutively expressed gene was used as reference gene (Sestili et al., 2014). All qRT-PCR reactions were carried out on a Rotor-Gene 6000 machine (Qiagen) with the following thermal cycling profile: 50°C for 2 min and 95°C for 2 min, followed by 40 cycles at 95°C for 3 s and 60°C for 30 s. Melting curve analysis was performed to verify single product amplification with temperature ranging from 55 to 95°C by increasing of 1°C every step. All reactions were performed in a total volume of 10 μl containing 30 ng of cDNA, 5 μl 1 × SYBR® Select Master Mix (Applied Biosystem) and 0.2 μl (20 μM) of each primer. For each sample, two biological replicates were analyzed in independent runs and a no-template control was included for each gene. Intra-assay variation was evaluated by performing all reactions in triplicate. The quantification cycle (Cq) was automatically determined using Rotor-Gene 6000 Series Software, version 1.7 as reported in Sestili et al. (2014).

**Table 1:** **Primer sequences for qRT-PCR validation of 10 melon sequences involved in defense responses and 3 fungal transcripts differently expressed in planta**.

**ID**	**Gene annotation**	**Fw_Left primer**	**Rv_Right primer**	**bp**
MELO3C002948	*NA*	CCAGTTCGGGTCAGGGAAAA	GCCTCGGGTTTAACAGTGGA	91
MELO3C010155	*NA*	CGACGATGAACACCACACAG	ATCTTCCGTCTGTCGTGCAG	81
MELO3C015129	*NA*	TCTGGCAAACGCTCTCCAAT	TTTTGCGAGCGTTTGGTGAG	142
MELO3C000909	*ANCHYRIN*	GGGTTAACAGCTCTTGACATCC	GGCGTCTGTGAGAGTCTCTG	81
MELO3C023962	*LRR RECEPTOR*	CGTTTGACCACCTTGAAGCG	TCTAACCCGATCCGGTTCCT	93
MELO3C017541	*LRR-SERINE/THREONINE KINASE*	TAATGGGTTTTCCGGGGAGC	TCGGAACCACACCTTCGAAG	92
MELO3C003144	*THAUMATIN LIKE PROTEIN*	ACACCAAAAGACGGGGGTTT	AGTTGCAGCCTTGTCTACCC	103
MELO3C017497	*PR1 E3-UBIQUITIN PROTEIN*	AGCAGCTGCAACTACCTCTG	CACACCAGGCGTTGTTTGAG	92
MELO3C007732	*LIGASE ATL6*	TGGAATGTGCCGTTTGCTTG	GTCGATGCAAGGGGGATGAA	95
MELO3C005291	*PECTINESTERASE*	CGCAGCGTTAACGGATCAAG	GCTTCTTCACCTTCTCCGCT	104
FOXG_06378	*FOW2*	CCAAGCTCTCCGTATCGCAT	CCCATCTGAGGCATGGTTGT	109
FOXG_00058	*FRP1*	CTGTTGAGACGCCGCAAAAA	ATTTGGAGGCAAGTCGGAGG	129
FOXG_01898	*SKP1*	TTGATGTCGGCTGCAAGACT	CCTCGGGAGTGAAGTCGTTC	99

### Mapping of RNA-Seq reads, DEG calling, and GO enrichment analyses

Raw fastQ files were checked for contaminants and low quality bases (quality <= 10 phred score) and contaminants were trimmed out with Cutadapt software (Martin, 2011). Contaminant-free, filtered reads were mapped with Tophat version 1.4.1 (Trapnell et al., 2012) to *C. melo* L. genome (CM version 3.5) (Garcia-Mas et al., 2012), furthermore, the same reads were mapped with tophat2 (Kim et al., 2013) against *F. oxysporum lycopersici* (Fol) genome (version F02.20, obtained from http://fungi.ensembl.org/Fusarium_oxysporum/Info/Index). For both genomes, raw read counts were obtained from BAM alignment files by counting with HTSeq software version 0.5.3 (Anders et al., 2015) in “union” mode. DESeq version 1.10.1 and DESeq2 version 1.2.8 (Love et al., 2014) Bioconductor packages were used to call melon and FOM1.2 DEGs, respectively. Both DESeq packages were developed implementing models based on negative binomial distribution with special attention to cope with biological variance. For the identification of melon DEGs (control vs. inoculated plants of both melon genotypes) the DESeq cutoff for considering a gene expressed was set to 0.5 RPKM, while the DESeq parameters for dispersion estimation were set with method “pooled” and sharing Mode “fitOnly.” The false discovery rate (FDR) threshold for DEG calling was set to 0.05. FOM1.2 DEGs were called by means of DESeq2 version 1.2.8 Bioconductor package. All genes with a log2 fold change (log2-fc) different from “NA” were considered as expressed. Parameters were set to fit type = “parametric,” BetaPrior = T, and independent filtering was allowed. The FDR threshold for DEG calling was set to 0.05. DESeq-normalized melon samples were transformed with function VST (DESEq package) and heatmaps were created with heatmap.2 (“gplots” Bioconductor package). The goseq bioconductor package (Robinson and Oshlack, 2010) was used to account for RNA length bias typical of RNA-Seq approaches (Oshlack and Wakefield, 2009). GO terms for melon genes were obtained by running BLAST2GO (Götz et al., 2008) using as query melon proteins (CM3.5.MELO.3C) against NR database, with the following annotation parameters: *E*-value hit filter 1.E-10, Annotation cut-off 55, GO weight 5, Hsp-Hit coverage cutoff 20. Gene reference sets were all genes above expression thresholds (RPKM > 0.5).

Read sequences have been uploaded at EBI (ArrayExpress), Accession Number: E-MTAB-4939.

### MapMan and KEGG tools

MapMan figures were obtained by running the Mercator tool (http://mapman.gabipd.org/web/guest/mercator) with default parameters to assign MapMan bins to melon transcripts (Thimm et al., 2004). Log2 fold changes as obtained from DESeq output were used as MapMan input to represent expression changes. The Bioconductor package Pathview version 1.6.0 (Luo and Brouwer, 2013) was used to generate the KEGG (Kyoto Encyclopedia of Genes and Genomes) pathway picture incorporating color-coded expression values. KEGG database integrates genomic information with higher order functional information by collecting manually drawn pathway maps representing current knowledge on cellular processes and standardized gene annotations (Kanehisa and Goto, 2000). Pathview parameters were set as default ones and the limit parameter were set as: limit = list (gene = 5, cpd = 1). As per Pathview default settings, log2-fc values for boxes representing more than one gene are summed up.

## Results

### Phenotypic screening of melon plants infected by FOM1.2

**Figure 1:**
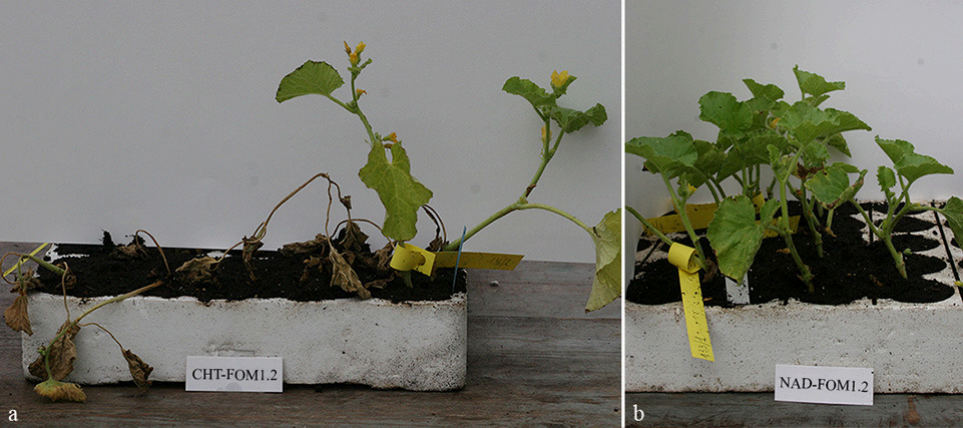
**Symptoms of FOM infection on the compatible (a)** and the incompatible **(b)** interactions at 21 and 35 dpi, respectively.

Fusarium wilt progression in the resistant DH line NAD and in the susceptible cultivar CHT, inoculated with FOM1.2, was monitored by a phenotypic screening at 8, 15, 21, and 35 dpi. The fusarium wilt symptoms as necrosis and severe lesions on plant collars became clearly evident in the compatible interaction already at 8 dpi. Due to the massive increase of fungal biomass in the xylem sap, 54 and 78% of CHT plants were dead at 15 and 21 dpi (Figure 1a), respectively. Despite the colonization of the tissues by fusarium, the NAD plants remained healthy and free of wilt symptoms during the time course experiment (35 dpi; Figure 1b), confirming the high level of resistance of this genotypes.

### RNA sequencing and identification of differentially expressed genes

**Figure 2:**
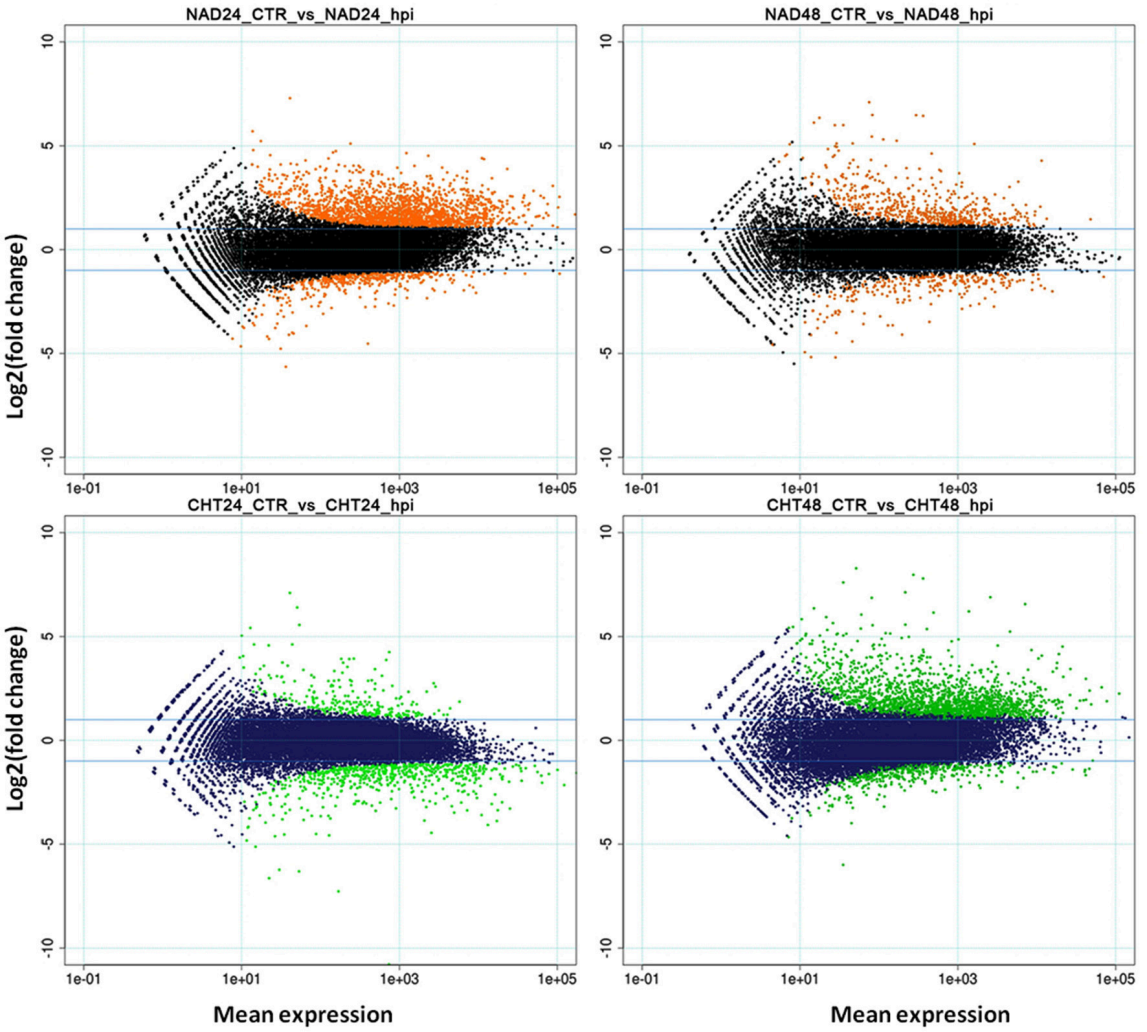
**MA-plots**. Mean expression (X-axis) vs. log2-fc (Y-axis) are represented. Orange **(upper panel)** and green **(lower panel)** dots represent DEGs in NAD and CHT, respectively, while black **(upper panel)** and blue **(lower panel)** dots represent non-modulated genes. CTR, control sample; hpi, hours post FOM1.2 inoculation.

RNA-Seq analysis was carried out on total RNA samples from both resistant (NAD) and susceptible (CHT) melon genotypes infected with FOM1.2 and mock in order to identify DEGs putatively involved in the resistance response. Illumina reads (51 bp) ranging from 12 to 25 millions/sample (on average 18.5 million reads) were obtained from the 16 samples (Supplementary Table 1). An RPKM cutoff value of 0.5 was set to declare a locus expressed, resulting in 19,162 and 18,615 *loci* above the expression cutoff for NAD and CHT, respectively. Pearson correlations between replicates were always above 0.9 and samples undergoing the same treatment clustered together (Supplementary Figure 1). The R package DESeq called 6,401 DEGs in total. NAD inoculated with FOM1.2 showed 2,461 and 821 DEGs at 24 and 48 hpi, respectively; among them 2,023 (82%) and 568 (69%) were up-regulated (Supplementary Table 2). The approximately three-fold ratio between DEGs at 24 and 48 dpi was reversed in the susceptible genotype CHT, where 882 and 2,237 DEGs were identified at 24 and 48 hpi, respectively; with only 255 (29%) up-regulated at 24 hpi, and 1,812 (81%) up-regulated at 48 hpi (Supplementary Table 3). The DEGs distribution patterns are illustrated in the MA plots (Figure 2). Notably, when all DEGs of NAD were compared to those of CHT only 79 genes were in common between the two genotypes.

Many melon unannotated transcripts (NA) were found to be differentially regulated after FOM1.2 infection and 1,486 were considered as “novel,” a class covering uncharacterized, hypothetical or predicted proteins and transcripts not reported in the RefSeq (MELO v3.5) gene annotations. In CHT, 254 and 542 NA DEGs were identified at 24 and 48 hpi, respectively, while in NAD 511 and 179 NA DEGs were recovered at 24 and 48 hpi, respectively. Among the NA DEGs identified in NAD at 24 hpi, 185 were regulated only in the resistant genotype and three of them (MELO3C002948, MELO3C010155, MELO3C015129) were validated by qRT-PCR since they were strongly induced, log2-fc > 2, and were not identified either in CHT or in the mock-inoculated samples (Supplementary Table 4).

### Gene ontology enrichment analysis

To point out the function of the genes involved in melon response to FOM1.2 infection, gene ontology (GO) enrichment analysis and MapMan overview of the metabolic pathways in both genotypes and time points were performed. In RNA-Seq experiments, GO enrichment analysis emphasizes that the status of modulated genes is related to the read counts and thus biases in favor of longer and highly expressed genes are expected (Young et al., 2010). To limit the bias in the data the GO enrichment was carried out *via* the Goseq R package (Young et al., 2010). Goseq output (threshold FDR = 0.05) yielded 27 enriched GOs shared by both the genotypes and time points. Among these the most relevant GO terms consistent with response to fungal infection were “defense response to fungus” (GO:0050832), “response to chitin” (GO:0010200), “plant-type cell wall” (GO:0009505), and “respiratory burst involved in defense response” (GO:0002679). However, several GO terms revealed to be genotype- and time-point-specific, disclosing substantial differences in the response of the two genotypes to FOM1.2 (Supplementary Figures 2–5). In NAD, the large part of the transcriptome modulated during FOM1.2 infection was devoted to defense mechanisms as revealed by 33 specific-GO terms enriched exclusively at 24 hpi, among them “defense response, incompatible interaction” (GO:0009814), “plant-type cell wall organization” (GO:0009664) “hyperosmotic response” (GO:0006972), “auxin polar transport” (GO:0009926), “cellulase activity” (GO:0008810) emerged as major components of the FOM1.2 resistance-associated response (Supplementary Figure 2). At 48 hpi, out of 18 NAD-specific GO terms, three, “oxylipin biosynthetic process” (GO:0031408), “response to jasmonic acid stimulus” (GO:0009753), and “calmodulin binding” (GO:0005516), deserved particular attention (Supplementary Figure 3). “Pectinesterase activity” (GO:0030599) and “manganese ion binding” (GO:0030145) were the only GO terms in common between the two time points of NAD-FOM1.2 combination, indicating that the presence of antioxidant enzymes is required to contrast the oxidative status due to the infection process (Supplementary Figures 2, 3). In CHT, the enriched GO terms were more abundant at 48 hpi than at 24 hpi (44 vs. 19) (Supplementary Figures 4, 5). At 24 hpi, “nitrate assimilation” (GO:0042128), “response to sucrose stimulus” (GO:0009744), and “photosynthesis” (GO:0015979) were the main GO terms enriched suggesting that the basic cell metabolism of CHT is modified in response to infection (Supplementary Figure 4). At 48 hpi, the plant metabolism switches to senescence pathways and triggers detoxification processes as confirmed by the presence of the “ethylene mediated signaling pathway” (GO:0009873), “ubiquitin-dependent protein catabolic process” (GO:0006511), and “glutathione metabolic process” (GO:0006749) GO terms (Supplementary Figure 5).

**Figure 3:**
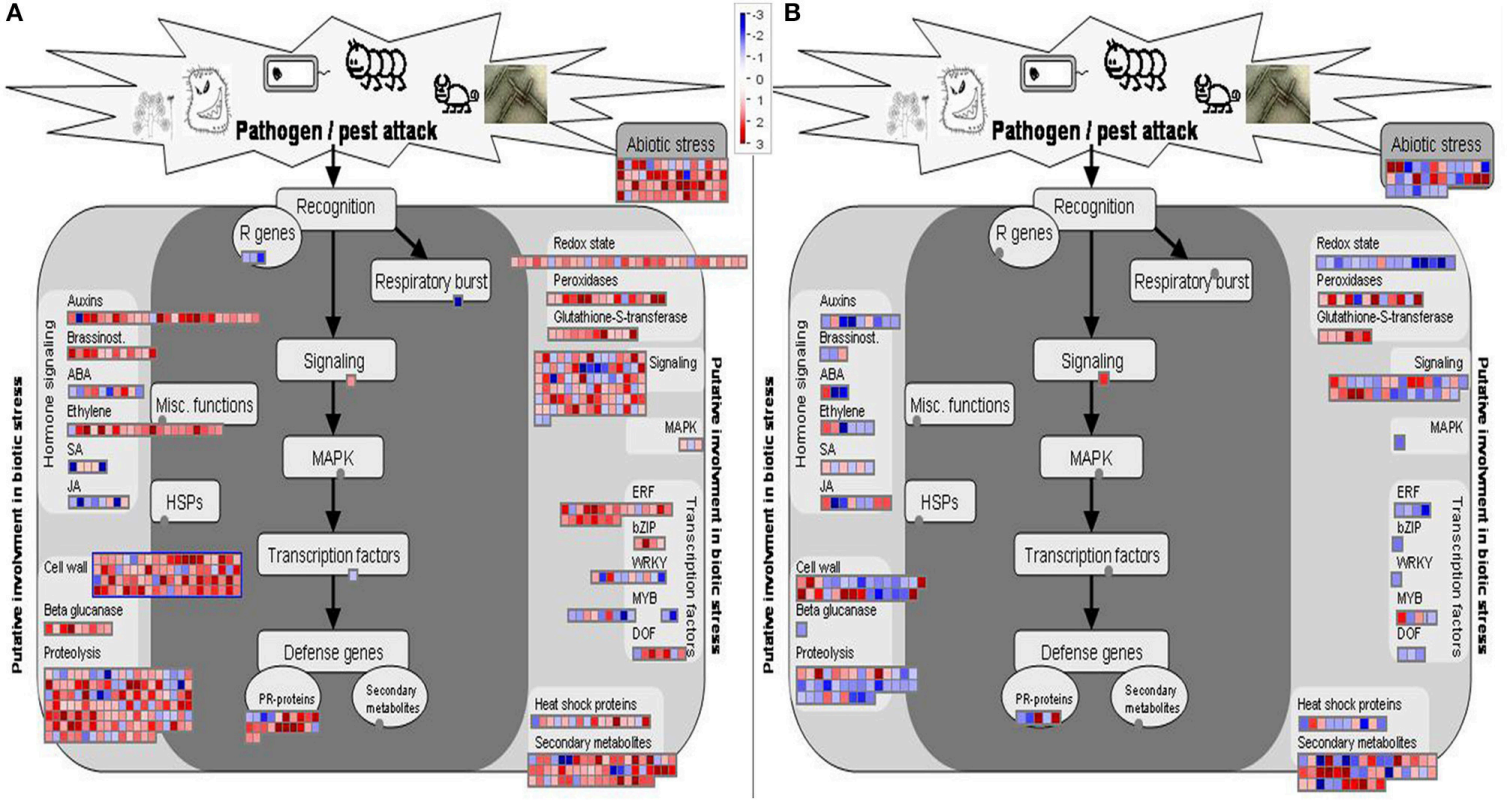
**MapMan overview (Biotic stress_panel) of the effect of FOM1.2 infection**. Expression changes on metabolism of resistant NAD **(A)** and susceptible CHT **(B)** melon plants at 24 hpi. Genes with experimental indication of involvement in the biotic stress are gathered on the main panel (colored with dark gray), while genes and pathways that are putatively involved in biotic stress pathway are shown on the left and right sides (colored in light gray). In both cases, the signal after infection is expressed as a ratio relative to the signal in uninfected controls, converted to a log2 scale, and displayed. Up-regulated and down-regulated transcripts are shown in red and blue, respectively. The scale is shown in the figures.

The transcriptional changes of resistant and susceptible melon seedlings were visualized via the MapMan software. Figures 3A,B show the general overview of metabolic changes and clearly confirm the opposite reaction of the two genotypes to FOM1.2 infection as already highlighted by the DEGs analysis. In NAD, the MapMan visualization reveals the regulation of 80 genes involved in the cell wall strengthening, 53 in transcription regulation, 60 in the oxidative respiratory burst, 92 in cellular signaling, and 22 genes related to PR proteins (Figure 3A). Furthermore, MapMan suggests the involvement of the hormone metabolism with 26 genes related to auxin, 10 to ABA, and 21 to ET (Figure 3A).

### Differentially expressed genes in response to FOM1.2 infection

**Figure 4:**
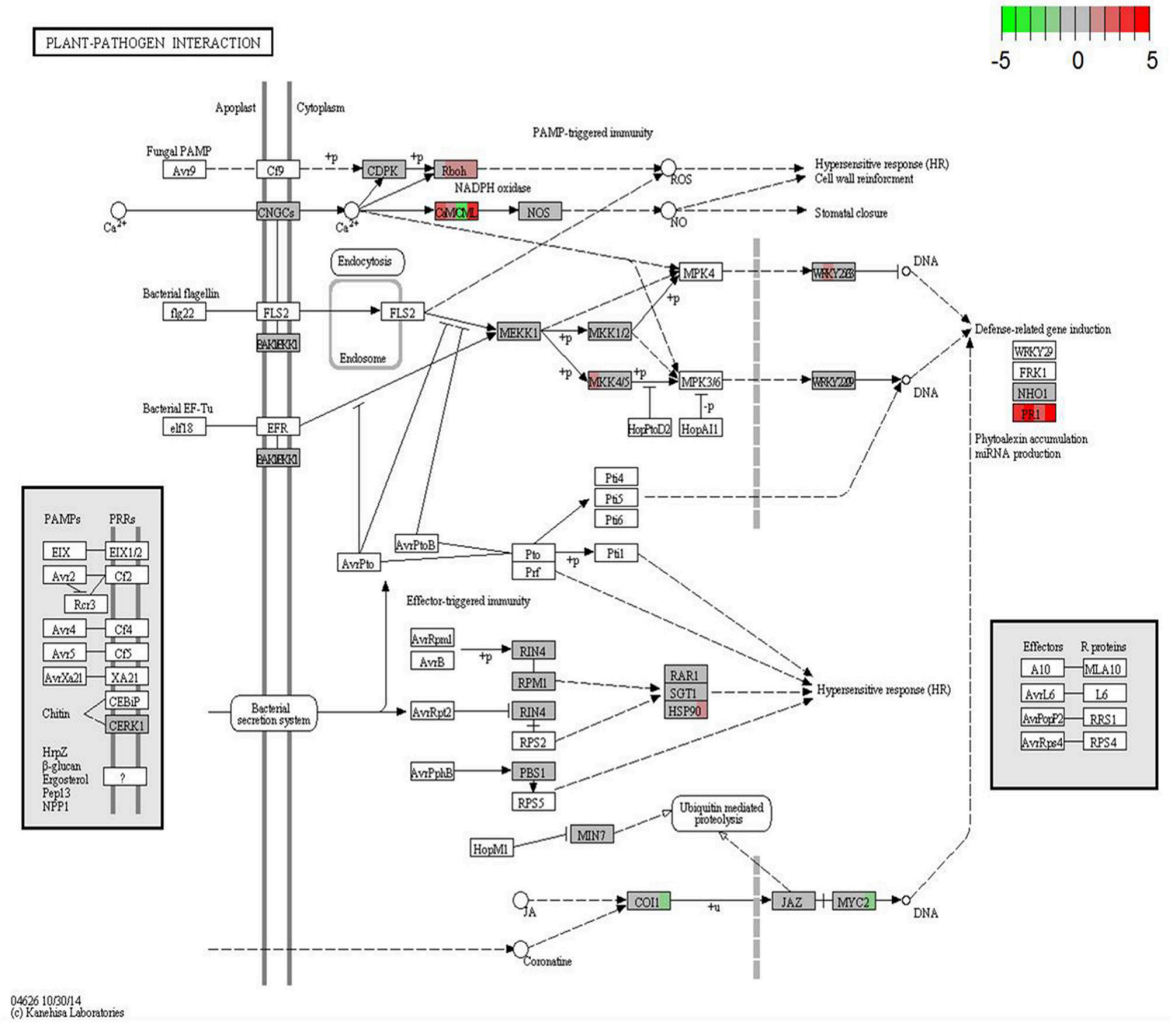
**KEGG orthology map (ko04626, plant-pathogen interaction) of melon-FOM1.2 pathosystem**. Colored boxes are placeholders for one or more genes as assigned to the same KEGG orthology group by KAAS server. For each box, from left to right, contrasts NAD24hpi_vs_NAD24CTR, NAD48hpi_vs_NAD48CTR, CHT24hpi_vs_CHT24CTR, and CHT24hpi_vs_CHT48CTR are depicted in color scale representing log2-fc values (infected over mock-inoculated) as indicated in color key, from −5 (sharp green) to +5 (sharp red). CTR, control sample; hpi, hours post FOM1.2 inoculation.

KEGG database (http://www.genome.jp/kegg/) was used to perform pathway mapping of the DEGs involved in melon-FOM1.2 interactions to facilitate the inspection of the plant and fungus networks and understand their overlapping and interactions. KEGG analysis revealed that unigenes where significantly enriched in various components involved in pathogen resistance mechanisms or signaling pathways (Figure 4).

#### Structural defense

The cell wall is the first line of active defense being involved in signal perception. Four *SUBTILISIN-LIKE PROTEASE* and 7 DEGs encoding for LEUCINE RICH-REPEAT (LRR) PROTEINS were specifically induced in the resistant plant in response to FOM1.2 (Supplementary Table 4). The up-regulation of *PR1* in NAD in the early phases of infection indicates that the degradation of the pathogen cell wall components is an important defense reaction in melon against FOM1.2 as revealed by KEGG enrichment analysis (Figure 4). Furthermore, the regulation of many genes responsible for cell wall reinforcement such as 5 *PROLINE-RICH GLYCOPROTEIN*, 7 *HYDROXYPROLINE-RICH GLYCOPROTEIN* (HRGP), 1 *CELLULOSE SYNTHASE*, 2 *SYNTAXIN* occurred in NAD (Supplementary Table 4) as shown also by MapMan (Figure 3). In addition, the NAD response to FOM1.2 was characterized by a strong induction of genes coding for polygalacturonase (PG), pectin methylesterase (PME), and pectin methylesterase inhibitor (PMEI) forming complex and pectin-degrading (pectate lyase) enzyme, all involved in the degradation of the cell wall (Supplementary Table 4). At 24 hpi, in NAD 5 *PECTINESTERASE INHIBITOR* and 3 *PECTINESTERASE* genes, probably PME+PMEI forming complex, were specifically co-expressed during pathogenesis (Supplementary Table 4).

#### Chemical defense

MapMan analysis revealed the induction of several genes encoding for proteins and secondary metabolites in NAD xylem sap at the early time point, i.e., *XYLEM SERINE PROTEINASE 1*, β*-1,3-GLUCANASES* (PR-2), *CHITINASES* (PR3), *THAUMATIN-LIKE PROTEINS* (TLPs) (PR5), and *PEROXIDASES* (PR-9) (Supplementary Table 4; Figure 3A). We identified five induced *CHITINASE* genes belonging to distinct classes (Supplementary Table 4), suggesting that an efficient chitinase activity might require a pool of diverse PR3 classes acting in concert. Eleven and 7 *PR3* genes were induced by FOM1.2 infection in NAD at 24 and 48 hpi, respectively (Supplementary Table 2), among them *MELO3C017677, MELO3C006704, MELO3C026772* are reputed to exert an effective defense role against FOM1.2 since they were expressed only at 24 hpi (Supplementary Table 4). Seven *TLP* genes were discovered in melon-FOM1.2 interaction, of which 6 were unique in NAD at 24 hpi (log2-fc > 1.75; Supplementary Table 4). The *MELO3C005642* gene was expressed in both genotypes, but dramatically up-regulated in NAD during the time course experiment (log2-fc from 7.3 to infinite; Supplementary Table 4). Furthermore, the *FLAVIN-CONTAIN MONOOXIGENASE1* (FMO1) gene was recovered only in NAD at 48 hpi (log2-fc = infinite; Supplementary Table 4). Several DEGs had an opposite regulation in the two genotypes showing late and/or missing response in CHT, as observed for 3 *PR* genes (MELO3C013762, MELO023694, and MELO3C010919) and for the *CHALCONE SYNTHASE* (CHS) gene (Supplementary Table 4). In NAD, *CHS* (*MELO3C014767*) was induced at 24 hpi (log2-fc = 2.13) (Supplementary Table 4); while, at the same time-point, the unique DEG coding for *CHS* (MELO3C010520) recovered in CHT was greatly down-regulated (log2-fc = −6.23), confirming its role in protecting plants against biotic stress. Among the defense related DEGs, the *E3 UBIQUITIN PROTEIN LIGASE ATL6 LIKE* gene was also specifically induced by FOM1.2 in NAD at 24 hpi (log2-fc = infinite; Supplementary Table 4).

#### Signaling

Three calmodulin (CaM) related DEGs induced only in the resistant plant during the infection were retrieved (Supplementary Table 4) suggesting the involvement of a Ca dependent signaling pathway. Furthermore, our findings confirm the key role of the hormones in melon defense against FOM1.2 as observed by the recovery of genes involved in salicylic acid (SA), jasmonate (JA), and ethylene (ET) pathways (Figure 3A). Among the SA-related genes, 5 DEGs encoding for novel “protein containing ankyrin repeats” were identified in NAD; among them only 2 were expressed along the experiment in CHT (Supplementary Table 4). A total of 30 DEGs related to ET responsive factors were recovered, including 10 *ETHYLENE-RESPONSIVE TRANSCRIPTION FACTOR-LIKE PROTEINS*, 15 *AP2 ERF DOMAIN CONTAINING TRANSCRIPTION FACTORS*, 3 *ETHYLENE-RESPONSIVE TRANSCRIPTION FACTOR ERF-LIKE*, 1 *ETHYLENE-RESPONSIVE TRANSCRIPTION FACTOR CRF4-LIKE*, and 1 *ETHYLENE-RESPONSIVE TRANSCRIPTION FACTOR RAP2-3-LIKE*; among them 19 DEGs were uniquely expressed by the resistant plant in response to FOM1.2 (Supplementary Table 4). In NAD, several JA-related genes such as *GERMIN-LIKE, PR4*, JA biosynthetic enzymes, and 4 LIPOXYGENASE (LOX) were induced and mainly regulated at 48 hpi (Supplementary Table 4). On the contrary, in CHT the JA signaling related genes were repressed resulting in lower activation of *CORONATINE INSENSITIVE 1* (COI1) and *MYC2* transcription factor genes (Figure 4). FOM1.2 infection also activates the transcription of auxin-related genes leading to a higher auxin biosynthesis. Seven auxin-related genes, i.e., 2 *AUXIN INFLUX CARRIER PROTEIN* (MELO3C013367; MELO3C003299), 1 *AUXIN INDUCED PROTEIN 5NG4-LIKE* (MELO3C003783), 1 *AUXIN RESPONSIVE FAMILY PROTEIN X15-LIKE* (MELO3C000885), 1 *AUXIN RESPONSIVE FAMILY PROTEIN 15A-LIKE* (MELO3C005991), and 2 *AUXIN RESPONSIVE PROTEINS IAA13-LIKE* (MELO3C023046; MELO3C006371), were exclusively induced in NAD at 24 hpi, suggesting their positive involvement in the resistance mechanism (Supplementary Table 4). Furthermore, in NAD the water stress induced by FOM1.2 is counteracted by the over expression of 5 abscissic acid (ABA)—receptors genes (Supplementary Table 4). In the NAD-FOM1.2 pathosystem, we observed the activation of mitogen-activated protein kinase (MAPK) cascade and WRKY transcription factor family members (Figure 4). In the resistant plant *MAPKK4* and *MAPKK5* genes (MELO3C002150, MELO3C025790), and 4 *WRKY* genes were induced at 24 hpi and *WRKY6* at 48 hpi (Supplementary Table 4). The MapMan analysis revealed the activation of the brassinosteroides pathways in the resistant genotype NAD, nevertheless, no genes known to be a significant pattern recognition receptor (e.g., the BRASSINOSTEROID INSENSITIVE 1-ASSOCIATED RECEPTOR KINASE 1) were recovered.

### Identification of FOM1.2 DEGs

The available FOM genomic sequence data are very limited; therefore, *Fol* genome was used as reference (version F02.20, obtained from http://fungi.ensembl.org/Fusarium_oxysporum/Info/Index). A total of 1,802 fungal DEGs were identified in the whole experiment, considering the mock inoculated samples as control. Among them, 728 and 322 DEGs were found in the CHT-FOM1.2 interaction at 24 and 48 hpi, respectively (Supplementary Table 5), while 143 and 609 DEGs were detected in the NAD-FOM1.2 combination at 24 and 48 hpi, respectively (Supplementary Table 6). In Supplementary Table 7 the selected FOM1.2 DEGs associated with pathogenicity are reported. The majority of FOM1.2 DEGs (58.7%) was assigned to the unannotated category, while among the annotated DEGs there was a significant presence of genes coding for cell wall degrading enzymes (CWDEs), cytoskeleton related protein, mitochondrial and vacuolar enzymes and fungal pathogenesis factors. Twenty-seven CWDE genes with 3.79 ≤ log2-fc ≤ 7.57 were identified in CHT-FOM1.2 interaction at 24 hpi, 14 of which resulted to be specific for either the compatible/incompatible interaction or the time-point (Supplementary Table 7). Among them, some deserve particular attention: 6 *ENDOGLUCANASE* (FOXG_02912, FOXG_04120, FOXG_05654, FOXG_07527, FOX_G09643, FOXG_13415); 2 *PECTIN*, and *PECTATE LYASE* precursor genes (FOXG_13249 and FOXG_16516); 1 *PG1* (FOXG_14695) and 3 *EXOPOLYGALACTURONASE* genes (FOXG_08862, FOXG_13191 and FOXG_15415). As shown in the Supplementary Table 7, the *ENDO-1,4-BETA-XYLANASE 2 PRECURSOR* gene (FOXG_09638) was considered as a putative pathogenicity factor because induced only in the susceptible plant. Instead, two *CHITIN SYNTHASE* genes, particularly *ChsVb* (FOXG_04163), virulence determinant in *F. oxysporum*, were induced to a higher extent in CHT and NAD at 24 and 48 hpi, respectively (Supplementary Table 7). The DEGs related to the cytoskeleton proteins *FIMBRIN* (FOXG_05565), *ACTIN* (FOXG_04579), and α*-* and ß*-TUBULIN* (FOXG_00655 and FOXG_06228) were mainly identified in the CHT-FOM1.2 interaction at 24 hpi (Supplementary Table 7). The high number of mitochondrial-related FOM1.2 DEGs, 72 and 43 in the compatible and incompatible interactions, respectively (Supplementary Tables 5, 6), suggests a substantial involvement of the mitochondrion in pathogenesis. The *FOW1* gene (FOXG_11292), encoding for a mitochondrial carrier protein, is constitutively expressed during the infection (Supplementary Table 7). Six selected vacuolar-related genes, i.e., *VACUOLAR ATP SYNTHASE SUBUNIT B* (FOXG_00954), *VACUOLAR ATP SYNTHASE SUBUNIT E* (FOXG_03408), *VACUOLAR PROTEIN SORTING-ASSOCIATED PROTEIN 21* (FOXG_09392), *VACUOLAR CALCIUM ION TRANSPORTER* (FOXG_03015), *VACUOLAR PROTEASE A PRECURSOR* (FOXG_12714), and *FVS1* (*F. oxysporum* VTS1 homolog encoding a SAM domain protein—FOXG_09534) were up-regulated only in the compatible interaction, mainly at 24 hpi, suggesting a dynamic vacuole trafficking during FOM1.2 infection (Supplementary Table 7). Another important virulence factor is the *FOW2* gene (FOXG_06378) that was more expressed in the CHT-FOM1.2 interaction at 24 hpi (log2-fc = 4.04) than in incompatible combination at 48 hpi (log2-fc = 3.79; Supplementary Table 7). Among the peroxisome related genes, 2 genes (*FOXG_09532* and *FOXG_17180*) expressed in compatible interaction at 24 with a high log2-fc, could be putative pathogenicity determinants (Supplementary Table 7). The SCF (Skp1, Cullins, and F-box proteins) E3 Ubiquitin ligase-mediated ubiquitin-proteasome system complex was also involved in the FOM1.2-melon interaction, the *FRP1* gene (FOXG_00058) was expressed at 24 hpi in the compatible and at 48 hpi in the incompatible interactions, while *SKP1* (FOXG_01898) was expressed only in compatible combination at 24 hpi (log2-fc = 3.70; Supplementary Table 7).

### Validation of DEGs by qRT-PCR

**Figure 5:**
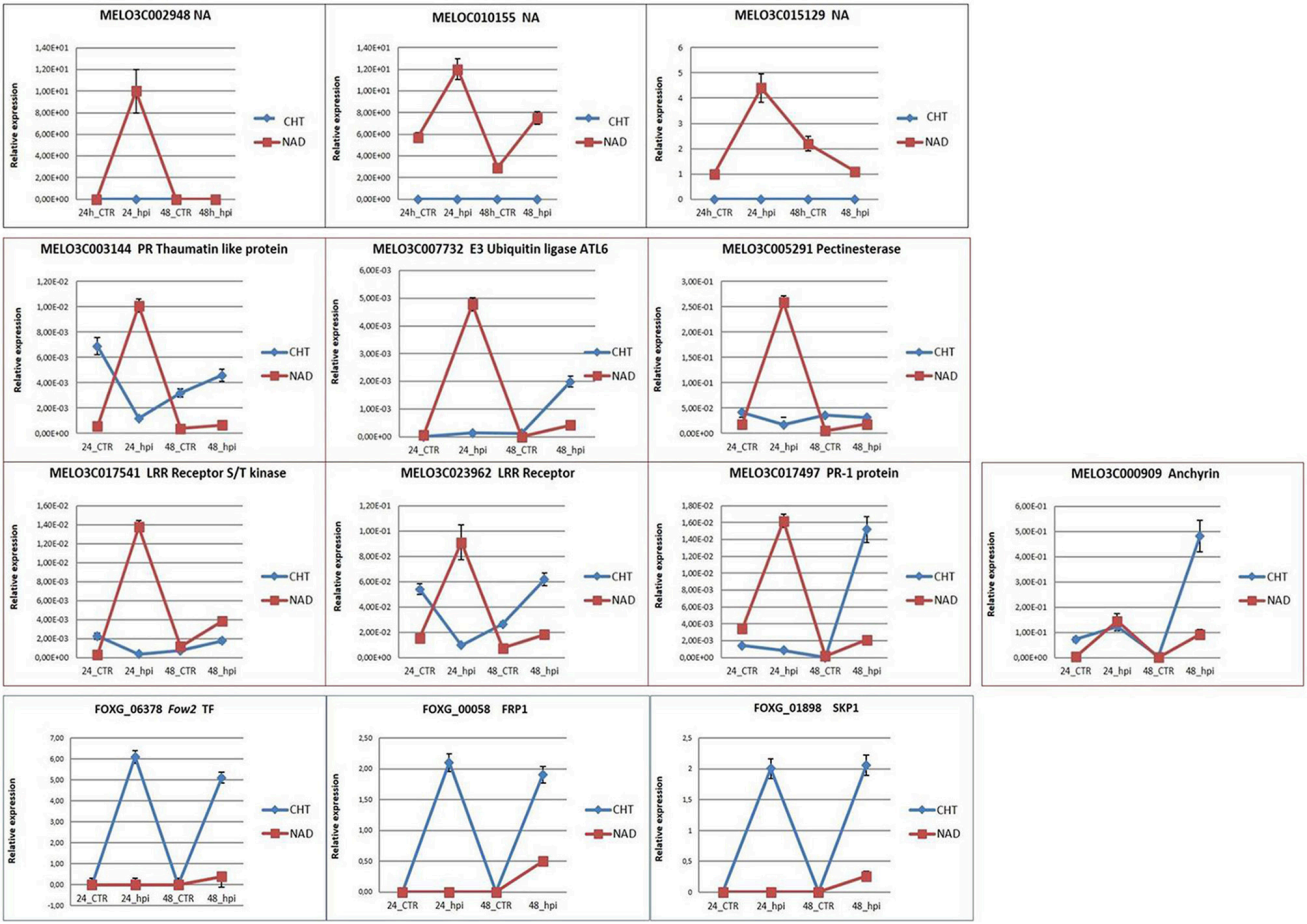
**qRT-PCR validation of the selected melon and fungal DEGs**. CTR, control samples; hpi, hours post FOM1.2 inoculation.

The qRT-PCR analysis was employed to validate the expression profiles of 10 melon and 3 fungal genes of particular interest (Table 1; Figure 5). Among the melon DEGs analyzed, 3 NA genes expressed only in NAD (*MELO3C002948, MELO3C010155, MELO3C015129*) were characterized by a similar trend of transcript accumulation, being activated at 24 hpi (log_2_-fc from 4.44 to 12.70) and repressed at 48 hpi (Figure 5) supporting the RNA-Seq data. The analysis of the other 6 melon genes (*TLP* MELO3C003144; *E3 UBIQUITIN LIGASE ATL6-LIKE* gene MELO3C007732; *PECTINESTERASE* gene MELO3C005291; *LRR RECEPTOR* genes MELO3C017541 and MELO3C023962; *PR1* gene MELO3C017497) showed a higher expression value at 24 hpi in NAD than in CHT (Figure 5) in good agreement with the RNA-Seq data. Only the qRT-PCR expression analysis of the *ANCHYRIN* gene, *MELO3C000909*, differed from the correspondent RNA-Seq data, showing a significant transcript accumulation in CHT at 48 hpi (Figure 5). The dissimilar expression levels between RNA-Seq and qRT-PCR could be caused either by whole-transcriptome data processing performed in the RNA-Seq analysis, presence of alternative transcripts not resolved by one of the two techniques, or by the dynamic nature of the transcriptome. The fungal *FOXG_06378, FOXG_00058, FOXG_01898* genes encoding for FOW2, FRP1, SKP1 resulted highly expressed in CHT along the whole experimental timeline, while in NAD they were slightly activated only at 48 hpi although with different log_2_-fc values (log_2_-fc from 2.00 to 6.12; Figure 5), these data support the results generated by RNA-Seq. As expected, these fungal transcripts were not recovered in the mock-inoculated samples. In conclusion, although the expression values were, in most cases, lower in RNA-Seq than in qRT-PCR, the obtained data confirmed the reliability of the results generated by RNA-Seq analysis.

## Discussion

### Melon-FOM1.2 interaction

The present study reports the first RNA-Seq analysis designed to described the changes in gene expression associated to susceptible and resistant melon-FOM1.2 interaction. Plant—*F. oxysporum* interaction has been studied in several species (Takken and Rep, 2010; Li et al., 2012; Zhu et al., 2013; Gordon and Koike, 2015). In melon, it is known that both the constitutive and inducible defense responses contribute to reduced FOM1.2 vascular colonization in resistant genotypes (Zvirin et al., 2010). In the resistant DH line NAD, the expression pattern of the genes critically involved in the resistance response highlights that the timely recognition of FOM1.2 invasion and the quick and effective induction of the defense mechanisms are the main traits associated with plant resistance. A previous work has demonstrated that the modulation of gene expression in responses to FOM occurs within the first 2 dpi and are maintained with few changes along the course of infection (Sestili et al., 2011b). FOM1.2 colonize the xylem vessels of both CHT and NAD plants although the fungal growth in compatible combination is higher, suggesting that NAD has the capacity of contrasting fungal development (Haegi et al., 2013). Many genes encoding defense-associated proteins, reinforcement cell-wall related genes, transcription factors, and signaling molecules involved in transduction pathways and production of reactive oxygen species (ROS) were recovered, in both genotypes, during the early melon defense events in response to FOM1.2 infection. In particular, the majority of DEGs related to primary innate immune system, i.e., *SUBTILISIN PROTEASES* and *LRR RECEPTOR* family members, were up-regulated only in NAD at 24 hpi. SUBTILISIN PROTEASE is an endogenous peptide known to be responsible for activating defense-related genes being available for receptor binding and initiation of defense signaling pathways (Figueiredo et al., 2014). Proteins with LRR motifs are involved in a different array of cellular processes; they provide an early warning system for the presence of potential pathogens and activate protective immune signaling acting as membrane bound signaling molecules (Figueiredo et al., 2014). The low number of expressed resistance genes (R-genes) found in our work is in agreement with the report of Garcia-Mas et al. (2012) which identified 411 putative disease R-genes in the melon genome out of which only 81 may exert disease resistance function as cytoplasmatic R-protein with NBS, tLRR, and tTIR domains. Similar results were found in banana, where the NBS-LRR-containing proteins were barely represented in the genome sequence (89 genes) compared to *Oryza sativa* (464 genes) and *Vitis vinifera* (459 genes; D'Hont et al., 2012). Furthermore, NAD promptly reacts to FOM1.2 penetration up-regulating many genes involved in cell wall fortification (e.g., *HRGPs* and *PEROXIDASES*) whose activity contribute to isolate the pathogen from the healthy host tissue. Notably, the same genes are repressed in the compatible CHT-FOM1.2 combination. A similar result was observed in banana plants infected with *F. oxysporum* f. sp. *cubense* (*Foc*) where plant resistance was driven by oxidative burst and cell wall lignification (Swarupa et al., 2014). CWDEs enable pathogen invasion of plant tissues although may trigger the plant defense response. Our data showed a strong expression of genes involved in polygalacturonase, pectinesterase, and pectin-degrading activity responsible for the degradation of the cell wall and playing a role in the response to pathogen attack. Plant polygalacturonase-inhibiting proteins (PGIPs) specifically bind and inhibit fungal PGs thus eliciting a variety of defense response induced by oligogalacturonides. The recovery of just one melon *PGIP* gene expressed in response to pathogen but with a similar expression profile in NAD and CHT does not support a key role of this gene in the resistance of NAD to FOM1.2 invasion. Studies on the defense roles of several *UBIQUITIN E3 LIGASES* provided clues about the regulation of pathogen-induced signaling (Berrocal-Lobo et al., 2010; Marino et al., 2012) since ubiquitination is known to play a critical role in the signaling pathways mediated by ABA (Stone, 2014). In response to FOM1.2 infection, NAD induces the expression of the *E3 UBIQUITIN PROTEIN LIGASE ATL6 like*-gene at 24 hpi. In *Arabidopsis*, the *ATL6* gene is rapidly up-regulated with the MAMPs elicitors and chitin as well as pathogen infection and control the abundance of the target protein 14-3-3 required for seedling response to carbon/nitrogen (C/N) stress and defense response (Maekawa et al., 2012; Stone, 2014). In tomato and pepper, E3 UBIQUITIN LIGASE (LeATL6 and CaRING1, respectively) plays a role in resistance to pathogens via the crosstalk between SA- and ET/JA-dependent pathways and is essential for timely activation of the hypersensitive response cell death (Hondo et al., 2007; Lee et al., 2011). Furthermore, the ATL6 mediated pathogen resistance, coordinated through MYB and WRKY transcription factors, was found to be responsible for both pathogen and C/N nutrient stress response (Stone, 2014). The emerged picture confirms the involvement of SA- and ET/JA-dependent pathways together with ABA signaling and the MYB and WRKY transcription factors in the resistance response to FOM1.2 infection in melon. Proteins as CHITINASES, TLPs, and PEROXIDASES as well as secondary metabolites that accumulate in the xylem sap during melon-FOM1.2 incompatible interaction at early time point could have an inhibitory effect on FOM1.2 virulence. CHITIN ELICITOR-BINDING PROTEIN, CHITIN ELICITOR RECEPTOR KINASE, and LysM RECEPTOR-LIKE KINASES resulted to be important components of the plant signaling cascades in response to FOM1.2 infection. Furthermore, the recovery of several *TLP* genes only in NAD at early time point and one expressed in both genotypes but strongly up-regulated in NAD confirmed the efficient response of the resistant plant against FOM1.2. *TLPs* are induced in response to pathogens attack and exhibit antifungal property when over-expressed in transgenic plants, probably by inhibiting hyphal growth, and spore germination (Acharya et al., 2013). The existence of calcium related-proteins in the plant nucleus, including CaM, CaM-binding protein, CDPK, and Ca^2+−^CaM-regulated protein phosphatase underline the involvement of calcium in signaling (Lecourieux et al., 2006). Recent evidence suggests that Ca^2+^ signaling *via* the up-regulation of CDPKs, CBL/CBL-interacting protein kinases and CaM may play a part in the melon-*Monosporascus cannonballus* interaction (Roig et al., 2012). Ca^2+^ signaling cascades and the increased cytosolic Ca^2+^ could represent an essential early event during melon-FOM1.2 interaction. In both NAD and CHT genotypes, the *CYCLIC NUCLEOTIDE GATED CHANNEL 2* gene, a possible candidate mediating the cytosolic calcium increase linking to nitric oxide (NO) production (Bellin et al., 2013), is expressed but not induced upon pathogen attack. In both genotypes, PAMPs triggered immunity is triggered by the RESPIRATORY BURST OXIDASE HOMOLOG and by CaMs proteins, earlier regulated in NAD, that activate the ROS and NO signaling pathways. The lack of effective pathogen sensing mechanism in CHT determines a delay in the activation of the calcium-signaling pathway leading to the disease, as also suggested by the recovery of some DEGs associated with calcium that were up-regulated only at 48 hpi. This result could identify Ca^2+^ as the pivotal intracellular messenger in the melon-FOM1.2 pathosystem confirming its role in triggering the biosynthesis of SA, JA, and ET and the oxidative burst that lead to the accumulation of ROS (Baluska, 2009; Xing et al., 2016). The disease outcome is often caused by the interactions among multiple hormone signaling pathways as ET, ABA, auxin, gibberellins, cytokinins, and brassinosteroids considered as hormone modulators of the SA–JA signaling backbone and key regulators of plant immunity (Pieterse et al., 2012). Although the crosstalk between the so-called “defense hormones” and “growth hormones” during plant defense is still largely unknown, these hormone pathways exhibit a key role in melon resistance to FOM1.2. The recovery of a limited number of DEGs related to SA in NAD and, on the other hand, a higher number in CHT at 48 hpi, suggested that SA appear not to be involved in the defense process as systemic resistance toward FOM1.2, but it may control JA and ET production during the stimulation of the defense. In NAD, this is confirmed by the higher and constant induction along the experiment of the SA-related genes such as the protein containing ankyrin repeats known to be regulators and effectors of defense response (Lu et al., 2003). When JA is bound to the F-box protein COI1 it leads to the degradation of JAZ protein family members and to the release of transcription factors such as MYC2, which promotes the expression of JA-responsive genes (Stotz et al., 2013). In the melon-FOM1.2 pathosystem the *COI1* and *MYC2* genes were down-regulated at 48 hpi in the susceptible combination, while no up-regulation was observed in the resistant plant confirming the partial involvement of JA-signaling pathway in the resistance response. In NAD, several LOX genes, involved in JA production, were induced at later stage of infection while the ET signaling genes (i.e., CRF, RAP, ERF) were early up-regulated; among them the ERF transcription factors, known to be involved in the regulation of JA-dependent defenses (Berrocal-Lobo and Molina, 2007), were the most retrieved. The recovery of high number of ET signaling genes in NAD at 24 hpi and a few in CHT at 48 hpi indicated the main and early involvement of ET pathways in the resistance response. In melon cotyledons, it has been reported that CELLULASES elicited by *Trichoderma longibrachiatum* induced ET and JA production, without accumulation of SA or hypersensitive response-like key reactions, speculating that two different pathways act in tandem to increase plant defenses (Martinez et al., 2001). Similar to SA and JA, auxin is a key signaling molecule regulating a wide range of growth and cellular processes associated with plants biotic stresses (Kazan and Manners, 2009). Both, the imbalances in auxin levels and changes in the expression of genes involved in auxin signaling foster pathogen infection (Bari and Jones, 2009). Our data suggest that FOM1.2 infection activates the transcription of auxin-related genes exclusively in NAD at 24 hpi leading to an increasing auxin biosynthesis and suggesting a positive involvement of auxin in the resistance mechanism. The last events of *Fusarium* infection are water stress, collapse of vascular vessels, necrosis and plant death (Di Pietro et al., 2003; Berrocal-Lobo and Molina, 2007). This critical status enhances pathogen invasion and could be responsible for the onset of the disease symptoms in CHT. Conversely, NAD is able to overcome this stressful condition by over-expressing ABA–related receptor genes such as *FMO1*, a gene expressed only in NAD in response to infection. FMOs, besides catalyzing specific steps in auxin biosynthesis, are known to have a role in pathogen defense. FMOs can detoxify compounds produced by the pathogens and modify the redox status by either influencing the intracellular glutathione levels or producing ROS (Schlaich, 2007). Our results indicate that auxin, ABA, and ET are the main hormones involved in NAD-FOM1.2 resistance similarly to what has been found in banana-*Foc* pathosystem (Li et al., 2012). The transcription factors and the genes involved in the signal transduction play a major role in tolerance and susceptibility inasmuch it has been observed that the susceptible plants are often defective in signal transduction pathways rather than in pathogen recognition mechanisms (Alves et al., 2013). NAD plants infected with FOM1.2 up-regulated MAPK cascade and WRKY family members following the chitin elicitor perception. In particular, two MAPKs related genes, *MAPKK4* and *MAPKK5*, were induced only in NAD at 24 hpi. Recently, a MAPK cascade involving MAPKK4, MAPK3 and MPK6 was shown to transduce chitin elicitor signal into defense responses in rice (Bagnaresi et al., 2012), and in *Arabidopsis* and tobacco, the activation of MAPKK4/MAPKK5-MAPKK6 cascade was observed to increase ET synthesis (Jakubowicz and Nowak, 2010). Our transcriptomic data indicate that the defense signaling pathways in melon-FOM1.2 pathosystem is likely dependent on *MAPKK4* and *MAPKK5*. Although, the SA pathway is only partially involved in FOM1.2 resistance in melon, the SA-related genes encoding for WRKY and PR-1 were induced only in the resistant plant. We speculate that the WRKYs could be activated by MAPK-mediated phosphorylation leading to an increased DNA binding activity. *WRKY12* and *WRKY6*, both involved in JA/ET-mediated signaling pathways, were the unique WRKY genes expressed only in NAD at 24 and 48 hpi, respectively. The late expression of WRKY6 confirmed the late activation of the JA signaling pathway in NAD as well as a possible nutrient-limitation stress during the infection. On the contrary, the *WRKY15* gene, involved in the ABA signaling, is the only *WRKY* expressed in both genotypes at 48 hpi, confirming its putative involvement in osmotic and oxidative stress response. The TGA transcription factor involved in SA-mediated defense (Lu, 2009) was not among the DEGs neither in the resistant nor in susceptible plants suggesting the main involvement of the ERF and WRKY signaling network in the resistance toward FOM1.2 in melon.

### Pathogen overview

For an in-depth understanding of the mechanisms involved in plant–pathogen interaction, the understanding of the in *planta* fungal gene modulation is an important prerequisite. In melon, FOM1.2 transcripts were predominantly detected during the late infection phase and almost exclusively in the susceptible plant. This is probably due to the presence of more fungal biomass in the susceptible plant as consequence of the pathogen invasion (Sestili et al., 2011b; Haegi et al., 2013). When a FOM1.2-GFP expressing strain was used to compare fungal colonization in susceptible CHT vs. resistant NAD melon genotypes the results have suggested that the fungus is able to grow in both susceptible and resistant plants (data not shown). All *Fusarium* genomes express a suite of CWDEs during the infection to gain access to nutrition, but very few genes of this class have been directly connected to pathogenicity (Aragona and Valente, 2013). The CWDE genes identified in melon-FOM1.2 pathosystem were mainly *ENDOGLUCANASES* known to be involved in fungus penetration and nutrient acquisition (Valente et al., 2011), *PECTIN* and *PECTATE LYASE* that degrade the pectic components of the plant cell wall, *PG1* gene that encodes the major *in vitro* extracellular ENDOPOLYGALACTURONASE (Benedetti et al., 2011) and 3 EXOPOLYGALACTURONASES that may have an important function in pathogen-plant interactions since they are generally not inhibited by plant PGIPs (García-Maceira et al., 2001). Our results confirmed that CHT is early colonized by the pathogen that proliferates and exerts its virulent action, while NAD triggers a quick defense response that contrast the FOM1.2 virulence and allow the plant to tolerate fungal growth. However, the ChsVb, virulence determinant in *F. oxysporum* and mediator of protection against plant defense compounds (Martín-Urdíroz et al., 2008), was induced to a high extent in CHT and NAD at 24 and 48 hpi, respectively. The identification of an *ENDO-1,4-BETA-XYLANASE 2 PRECURSOR* gene induced only in CHT-FOM1.2 combination, suggested that the induction of this gene is not peculiar to FOM race 1 as reported by Sestili et al. (2011b). Genes related to cytoskeleton components were predominantly identified in CHT-FOM1.2 combination at 24 hpi, confirming that the rapid cytoskeleton rearrangement is of crucial importance for pathogenicity. An important pathogenicity factor, essential for the colonization of plant tissue and virulence of *F. oxysporum*, is FOW1 that caused marked reduction of virulence in tomato (Inoue et al., 2002). In our pathosystem, the FOW1 gene was not differentially expressed during the time course of the experiment, although the high number of mitochondrial-related DEGs suggests a substantial involvement of the mitochondrion in FOM1.2 pathogenesis. Another important role in FOM1.2 pathogenicity is played by the vacuolar related DEGs and the *FVS1* gene deserves particular attention since FVS1-disrupted mutant from the melon wilt pathogen FOM led to defective conidial germination and virulence (Iida et al., 2014). In the susceptible plant (CHT), the FOW2 virulence factor, that encodes a Zn(II)2Cys6-type transcription regulator, controlling the competency of *F. oxysporum* was quickly expressed. The analysis of fungus DEGs in *planta* suggested that in the resistant plant NAD, the FRP1/SKP1 interaction is probably lacking. *FRP1*, an important pathogenicity gene that regulate the presence of CWDEs allowing fusarium wilt pathogen to enter the host xylem tissue (Sutherland et al., 2013), is present only at 48 hpi while SKP1 was never recovered in NAD. FRP1 interacts with SKP1, a component of the ubiquitin SCF complex that regulate a variety of cellular functions including virulence, and codes for a PR1-like protein constitutively expressed during infection and under a variety of culture conditions (Prados-Rosales et al., 2012). Thus, the lack of the interaction between these two pathogenicity genes could led to a weakening of FOM1.2 virulence in NAD.

## Conclusions

In the present study, the whole transcriptomes of compatible and incompatible melon-FOM1.2 interactions were characterized by RNA-Seq analysis at 24 and 48 hpi. The differences in terms of DEGs between the resistant line NAD and the susceptible cultivar CHT were more evident at the early stage of infection, confirming the prompt activation of the defense responses in NAD. The induction of the defense pathways requires the generation of endogenous signaling molecules at the elicited site. The first defense line is represented by CWDEs, but NAD defense against FOM1.2 was mainly signaled by JA/ET and ABA, which act in tandem to induce local and systemic expression of defense genes. In melon, the ET-mediated signaling pathway was activated earlier than the JA-mediated networks and the ET related genes were probably activated by MAPKK4/5. Furthermore, auxin emerged as the hormone that mainly contributed to the defense reaction in melon-FOM1.2 pathosystem. Apart from plant endogenous elicitors that trigger or amplify the immune response, pathogen-derived elicitors that can activate the plant innate immune response have also been identified. Our results demonstrate that NAD, in response to FOM1.2 infection, utilizes different, effective defense pathways comprising a complex resistance network, and suggest that these signaling pathways are not independent, instead interacting. Further investigations will be focused on functional validation and mapping of the selected DEGs, which could represent a helpful tool for developing melon resistant varieties toward FOM1.2.

## Author contributions

MS, SS, LC, NF conceived and designed the experiments; NF provided all the genetic materials. MS, SS, NF carried out the artificial inoculations. MS, CB performed the RNA extraction and library preparations. PB, AZ performed bioinformatics analyses. LO carried out the sequencing. MS, SS carried out data analysis and drafted the manuscript. LC, NF supervised the experiments and revised the manuscript. All authors read and approved the final manuscript.

### Conflict of interest statement

The authors declare that the research was conducted in the absence of any commercial or financial relationships that could be construed as a potential conflict of interest.

## Supplementary material

Supplementary Table 1 **Read and alignment data**.

Supplementary Table 2 **Complete list of NAD DEGs regulated by infection with FOM1.2 at 24 and 48 hpi, as defined in the results section**.

Supplementary Table 3 **Complete list of CHT DEGs regulated by infection with FOM1.2 at 24 and 48 hpi, as defined in the Results Section**.

Supplementary Table 4 **List of selected melon DEGs after FOM1.2 infection**.

Supplementary Table 5 **Complete list of FOM1.2 DEGs recovered in CHT at 24 and 48 hpi, as defined in the Results Section**.

Supplementary Table 6 **Complete list of FOM1.2 DEGs recovered in NAD at 24 and 48 hpi, as defined in the Results Section**.

Supplementary Table 7 **List of selected FOM1.2 DEGs in melon**.

Supplementary Figure 1 **Hierarchical clustering of samples**. Pearson correlations between biological replicates of resistant NAD **(A)** and susceptible CHT **(B)** melon plants. The heatmap was generated starting from DESeq normalized counts and following data transformation via variance stabilizing transformation function (DESeq package). R, biological replicate sample; CTR, control sample; hpi, hours post FOM1.2 inoculation.

Supplementary Figure 2 **GO terms specifically enriched in NAD at 24 hpi but not in CHT at 24 hpi**. The GO ontologies are split into cellular component (green bars), molecular function (blue bars), and biological process (red bars). hpi, hours post FOM1.2 inoculation.

Supplementary Figure 3 **GO terms specifically enriched in NAD at 48 hpi but not in CHT at 48 hpi**. The GO ontologies are split into cellular component (green bars), molecular function (blue bars), and biological process (red bars). hpi, hours post FOM1.2 inoculation.

Supplementary Figure 4 **GO terms specifically enriched in CHT at 24 hpi but not in NAD at 24 hpi**. The GO ontologies are split into cellular component (green bars), molecular function (blue bars), and biological process (red bars). hpi, hours post FOM1.2 inoculation.

Supplementary Figure 5 **GO terms specifically enriched in CHT at 48 hpi but not in NAD at 48 hpi**. The GO ontologies are split into cellular component (green bars), molecular function (blue bars), and biological process (red bars). hpi, hours post FOM1.2 inoculation.

## Abbreviations

ABA: abscisic acid
CaM: calmodulin
CHS: chalcone synthase
CHT: Charentais-T
C/N: carbon/niotrogen
COI1: coronatine insensitive 1
Cq: quantification cycle
CWDEs: cell wall degrading enzymes
DEGs: differentially expressed genes
DH: doubled-haploid
dpi: days post inoculation
ET: ethylene
FDR: false discovery rate
FMO1: flavin-contain monooxigenase1
Foc: *Fusarium oxysporum* f. sp. Cubense
Fol: *Fusarium oxysporum* f. sp. Lycopersici
FOM1.2: *Fusarium oxysporum* f. sp. melonis race 1.2
GO: Gene ontology
hpi: hours post FOM1.2 inoculation
HRGP: hydroxyproline-rich glycoprotein
JA: jasmonic acid
KEGG: Kyoto Encyclopedia of Genes and Genomes
log2-fc: log2 fold change
LOX: lipoxygenase
LRR: leucine rich-repeat
MAPK: mitogen-activated protein kinase
NA: unannotated transcripts
NO: nitric oxide
PG: polygalacturonase
PGIPs: polygalacturonase-inhibiting proteins
PME: pectin methylesterase
PMEI: pectin methylesterase inhibitor
R-genes: Resistance genes
RNA-Seq: RNA-Sequencing
ROS: reactive oxygen species
SA: salicylic acid
SCF: Skp1–Cullin–F-box protein
TLPs: thaumatin-like proteins.
