# Corrigendum: Transcriptome Analysis of the Melon-*Fusarium oxysporum* f. sp. *melonis* Race 1.2 Pathosystem in Susceptible and Resistant Plants

**DOI:** 10.3389/fpls.2017.00922

**Published:** 2017-05-30

**Authors:** M. Silvia Sebastiani, Paolo Bagnaresi, Sara Sestili, Chiara Biselli, Antonella Zechini, Luigi Orrù, Luigi Cattivelli, Nadia Ficcadenti

**Affiliations:** ^1^Research Unit for Vegetable Crops in Central Areas, Council for Agricultural Research and EconomicsAscoli Piceno, Italy; ^2^Genomics Research Centre, Council for Agricultural Research and EconomicsPiacenza, Italy

**Keywords:** RNA-Seq, plant–pathogen interaction, gene expression, Fusarium wilt, melon, molecular mechanism

In the original article, there was an error. The mistake concerns the citation of the code (identity) of the isolate of *Fusarium oxysporum* f. sp. *melonis* (FOM) Race 1.2 used in the work. The correct isolate code is: ISPaVe1680 (the code is mentioned just once in Materials and Methods Section - Plant Material and *In vivo* Inoculations). The authors apologize for this error and state that this does not change the scientific conclusions of the article in any way.

## Conflict of interest statement

The authors declare that the research was conducted in the absence of any commercial or financial relationships that could be construed as a potential conflict of interest.

