# Supplementary material for: Transcriptome Analysis of the Melon-Fusarium oxysporum f. sp. melonis Race 1.2 Pathosystem in Susceptible and Resistant Plants
Source: Front Plant Sci. 2017 Mar 17;8:362. doi: 10.3389/fpls.2017.00362 (PMC5356040; doi:10.3389/fpls.2017.00362)
Supplement: Supplementary Table 1 — Read and alignment data. [file Table1.DOCX]

**Supplementary Table 1. Read and alignment data.**

| **Samples** | **Illumina passed-filter** | **Contaminant free** | **Total alignments** | **Mapped reads** | **Uniquely mapped reads** |
| --- | --- | --- | --- | --- | --- |
| NAD_control_24h_1R | 19,181,676 | 18,871,022 | 19,155,684 | 18,223,397 | 17,589,670 |
| NAD_control_24h_2R | 20,514,891 | 20,284,245 | 20,255,130 | 19,570,795 | 19,108,335 |
| NAD_infected_24h_1R | 22,110,709 | 21,750,829 | 21,815,220 | 20,602,921 | 19,876,896 |
| NAD_infected_24h_2R | 19,877,440 | 19,436,342 | 19,374,115 | 18,531,325 | 17,962,969 |
| NAD_control_48h_1R | 18,764,296 | 18,496,988 | 18,266,326 | 17,818,229 | 17,511,180 |
| NAD_control_48h_2R | 15,766,584 | 15,400,859 | 15,301,821 | 14,829,214 | 14,505,549 |
| NAD_infected_48h_2R | 20,355,344 | 20,020,385 | 19,661,240 | 19,166,623 | 18,815,392 |
| NAD_infected_48h_2R | 25,353,337 | 24,743,002 | 24,466,362 | 23,626,737 | 23,010,080 |
| CHT_control_24h_1R | 16,723,007 | 16,393,253 | 17,186,276 | 15,366,305 | 14,254,086 |
| CHT_control_24h_2R | 16,483,206 | 16,280,414 | 16,263,182 | 15,620,871 | 15,194,697 |
| CHT_infected_24h_1R | 14,620,721 | 13,970,663 | 13,684,940 | 13,317,799 | 13,061,691 |
| CHT_infected_24h_2R | 16,750,447 | 16,541,183 | 16,330,573 | 15,915,948 | 15,625,369 |
| CHT_control_48h_1R | 16,741,386 | 16,586,085 | 16,393,568 | 16,010,067 | 15,746,764 |
| CHT_control_48h_2R | 22,366,924 | 22,134,163 | 21,937,793 | 21,390,345 | 21,025,848 |
| CHT_infected_48h_1R | 12,888,107 | 12,271,022 | 12,457,799 | 11,345,070 | 10,639,496 |
| CHT_infected_48h_2R | 16,750,542 | 16,447,943 | 16,105,086 | 15,659,318 | 15,358,087 |
