# Supplementary figures and images for: Transcriptome Analysis of the Melon-Fusarium oxysporum f. sp. melonis Race 1.2 Pathosystem in Susceptible and Resistant Plants
Source: Front Plant Sci. 2017 Mar 17;8:362. doi: 10.3389/fpls.2017.00362 (PMC5356040; doi:10.3389/fpls.2017.00362)

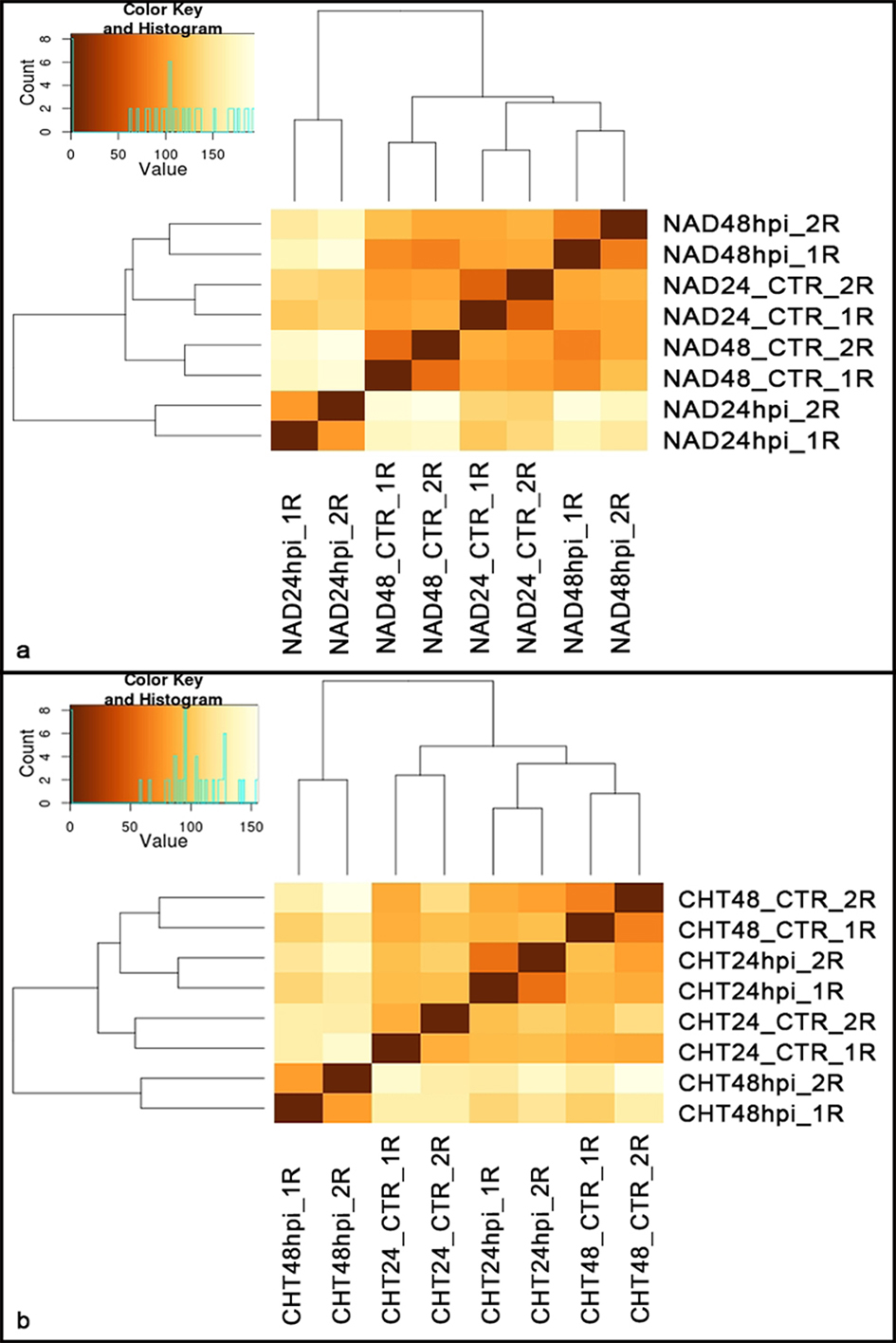

Supplement: Supplementary Figure 1 — Hierarchical clustering of samples. Pearson correlations between biological replicates of resistant NAD (A) and susceptible CHT (B) melon plants. The heatmap was generated starting from DESeq normalized counts and following data transformation via variance stabilizing transformation function (DESeq package). R, biological replicate sample; CTR, control sample; hpi, hours post FOM1.2 inoculation. [file Image1.JPEG]

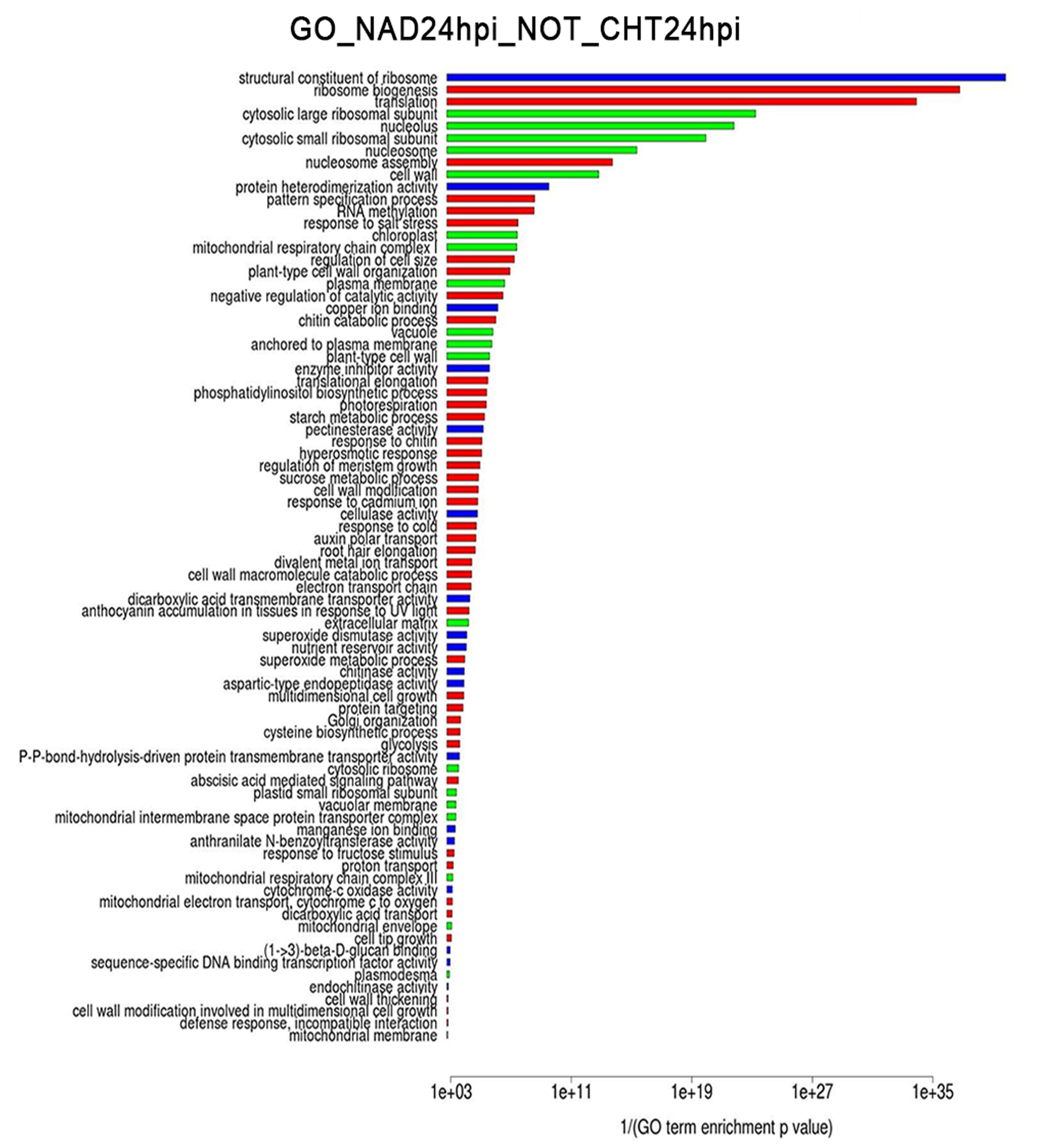

Supplement: Supplementary Figure 2 — GO terms specifically enriched in NAD at 24 hpi but not in CHT at 24 hpi. The GO ontologies are split into cellular component (green bars), molecular function (blue bars), and biological process (red bars). hpi, hours post FOM1.2 inoculation. [file Image2.JPEG]

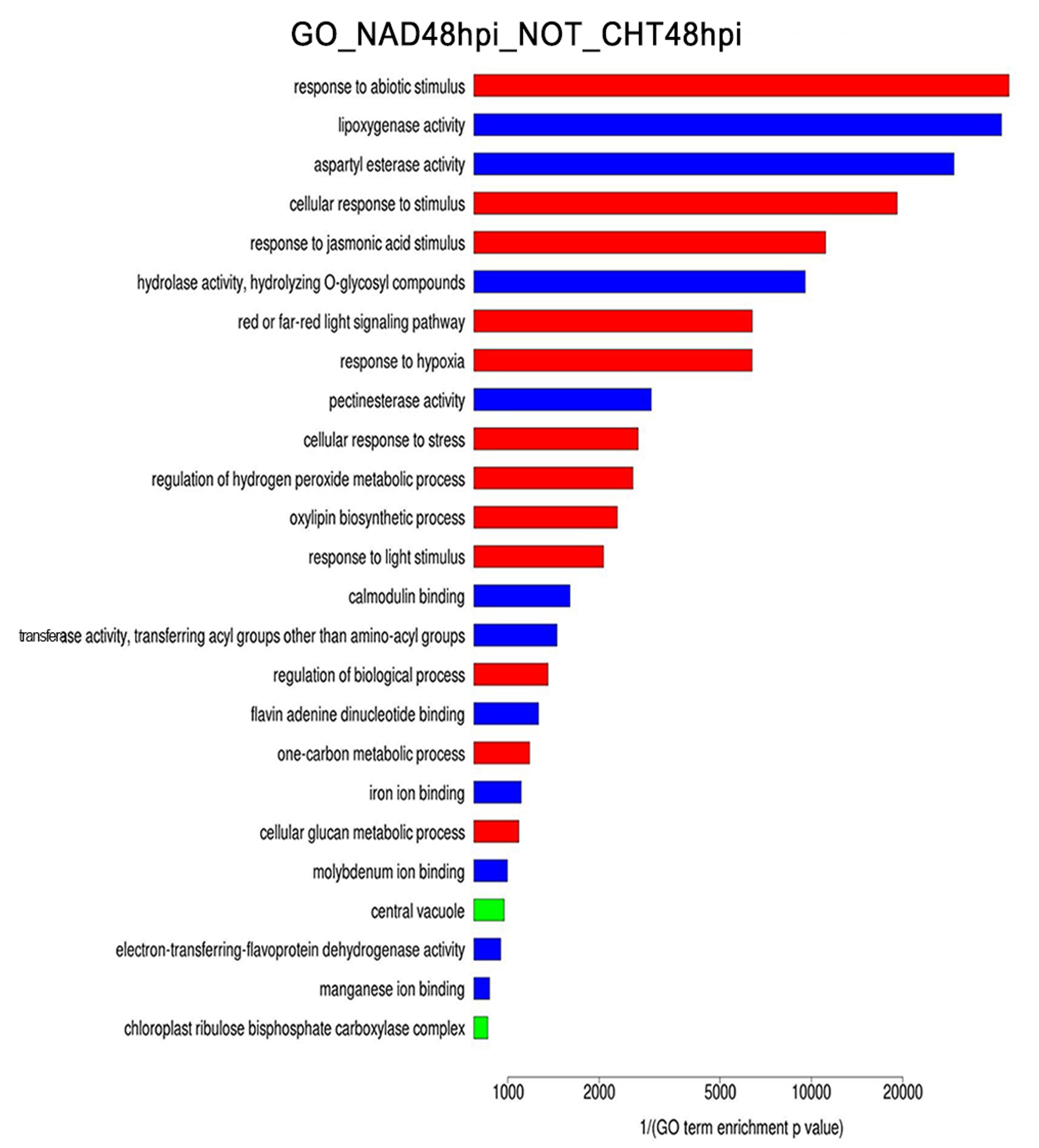

Supplement: Supplementary Figure 3 — GO terms specifically enriched in NAD at 48 hpi but not in CHT at 48 hpi. The GO ontologies are split into cellular component (green bars), molecular function (blue bars), and biological process (red bars). hpi, hours post FOM1.2 inoculation. [file Image3.JPEG]

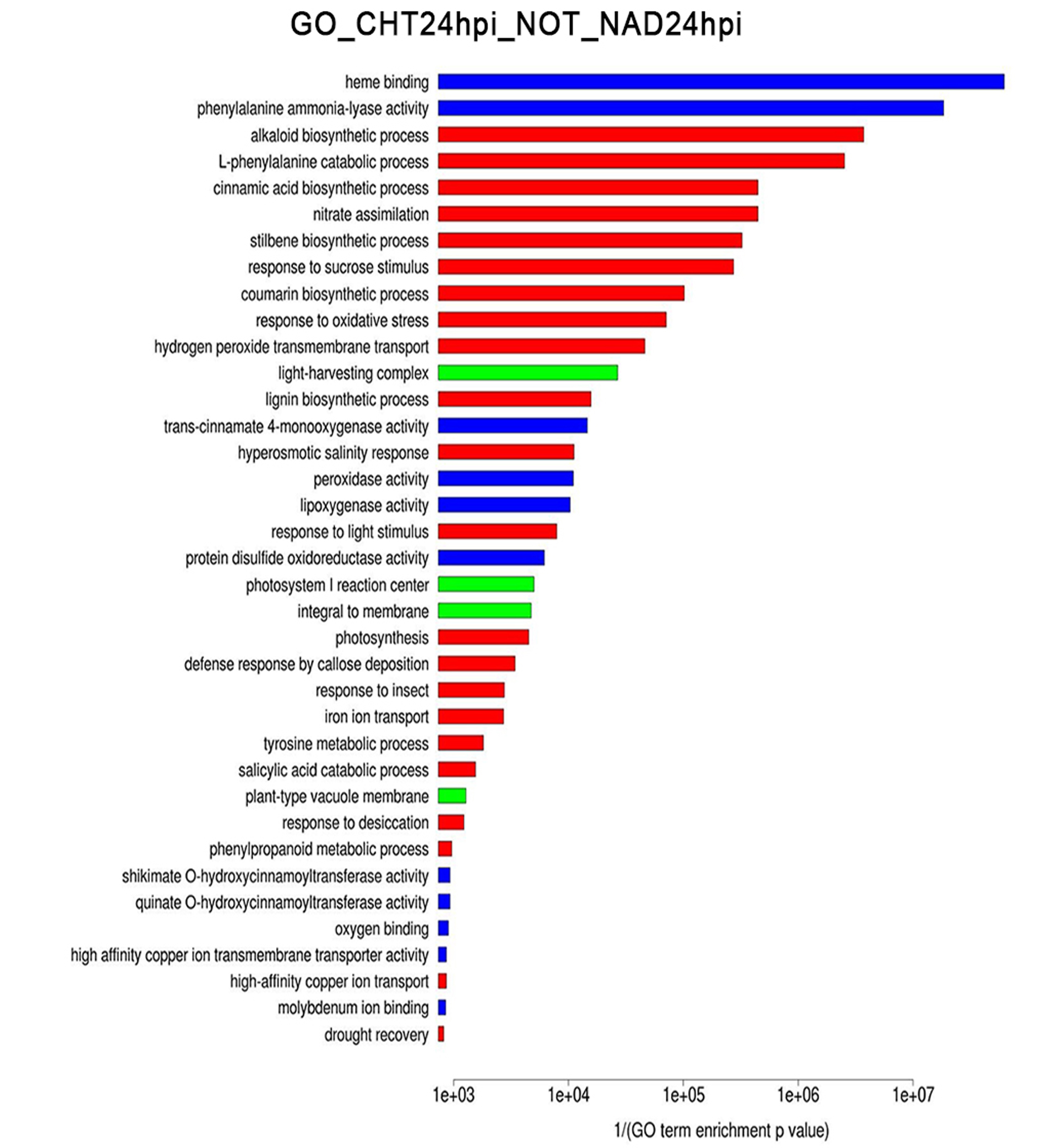

Supplement: Supplementary Figure 4 — GO terms specifically enriched in CHT at 24 hpi but not in NAD at 24 hpi. The GO ontologies are split into cellular component (green bars), molecular function (blue bars), and biological process (red bars). hpi, hours post FOM1.2 inoculation. [file Image4.JPEG]

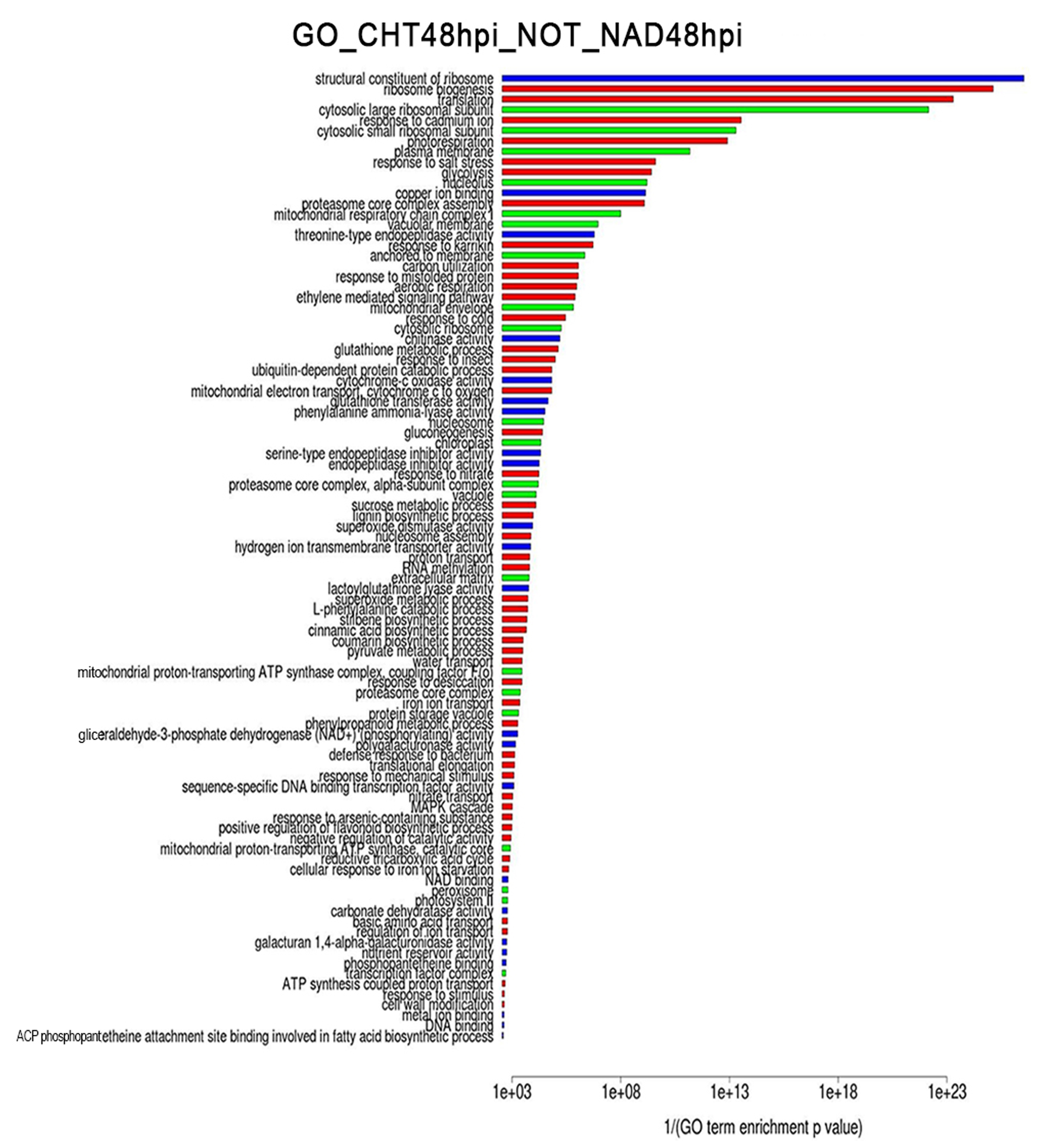

Supplement: Supplementary Figure 5 — GO terms specifically enriched in CHT at 48 hpi but not in NAD at 48 hpi. The GO ontologies are split into cellular component (green bars), molecular function (blue bars), and biological process (red bars). hpi, hours post FOM1.2 inoculation. [file Image5.JPEG]
